# Intra and inter-rater repeatability of brachial artery ultrasound estimates of flow-mediated slowing and flow-mediated dilation

**DOI:** 10.1371/journal.pone.0287759

**Published:** 2023-06-28

**Authors:** João Luís Marôco, Tiago Silvestre, Inês Arrais, Marco Pinto, Helena Santa-Clara, Bo Fernhall, Xavier Melo

**Affiliations:** 1 Integrative Human Physiology Laboratory, Manning College of Nursing & Health Sciences, University of Massachusetts Boston, Boston, MA, United States of America; 2 Ginásio Clube Português, Research & Development Department, GCP Lab, Lisboa, Portugal; 3 Centro Interdisciplinar de Estudo da Performance Humana (CIPER), Faculdade de Motricidade Humana, Universidade de Lisboa, Oeiras, Portugal; 4 Faculdade de Ciências da Saúde e do Desporto, Universidade Europeia, Lisboa, Portugal; 5 Center for Sports Optimization (COD), Sporting Clube de Portugal, Lisbon, Portugal; 6 Centro de Investigação Interdisciplinar Egas Moniz (CiiEM), Egas Moniz School of Health & Science, Caparica, Almada, Portugal; University of Perugia, ITALY

## Abstract

Flow-mediated slowing (FMS) is a non-invasive measure of endothelial function measured through reactive hyperemia-induced changes in pulse wave velocity (PWV). FMS is suggested to mitigate known pitfalls of flow-mediated dilation (FMD) including suboptimal repeatability and high-operator dependency. However, the few single-rater studies that examined FMS repeatability have shown controversial results and used only regional measurements of PWV, which might not reflect local brachial artery stiffness responses to reactive hyperemia. We assessed the inter- and intra-rater repeatability of ultrasound-based changes in local PWV (FMS) and diameter (FMD). Twenty-four healthy male participants aged 23–75 yr, were examined on two separate days. Reactive hyperemia-induced changes in PWV were calculated using a tailored R-script. The inter- and intra-rater repeatability were tested with the intraclass correlation coefficient (ICC), coefficient of variation (CV), and the Bland-Altman plot estimates. The inter-rater repeatability of FMS (bias: -0.08%; ICC: 0.85; 95% CI: 0.65 to 0.93; CV: 11%) and FMD (bias: -0.02%; ICC: 0.98; 95% CI: 0.97 to 0.99; CV: 7%) showed overall good repeatability over different days. The intra-rater repeatability of FMD (1^st^ rater: bias: 0.27%; ICC: 0.90; 95% CI: 0.78 to 0.96; CV: 14%; 2^nd^ rater: bias: 0.60%; ICC: 0.85; 95% CI: 0.64 to 0.94; CV: 18%) was better than FMS (1^st^ rater: bias: -1.03%; ICC: 0.76; 95% CI: 0.44 to 0.91; CV: 21%; 2^nd^ rater: bias:-0.49%; ICC: 0.70; 95% CI: 0.34 to 0.80; CV: 23%) but not different between raters. Ultrasound-based local measurements of PWV deceleration reactive hyperemia were repeatable among the raters.

## Introduction

Endothelial dysfunction is characterized by reduced nitric oxide (NO) bioavailability, increased permeability to plasma lipoproteins, and adhesiveness to leukocytes [1]. The assessment of endothelial dysfunction is of clinical relevance as it precedes clinical manifestations of atherosclerosis [1, 2], and independently predicts cardiovascular and all-cause mortality [3, 4].

Brachial artery flow-mediated dilation (FMD) is the most widely used non-invasive method to quantify endothelial function [5, 6]. However, FMD remains to be used in clinical settings given its high operator dependency and suboptimal repeatability. (CV > 10%) [7–9]. To overcome these limitations, flow-mediated slowing (FMS) has emerged as a methodological non-invasive alternative to indirectly access endothelial function through reactive hyperemia-induced changes in regional pulse wave velocity (PWV)–a surrogate marker of arterial stiffness [10, 11]. Reactive hyperemia is expected to reduce PWV through NO-mediated vasodilation, as vessel diameters are inversely proportional to PWV based on the Moens–Korteweg equation [12–14]. Seminal research showed that regional FMS exhibits better repeatability (CV: 7%) compared to FMD (CV:27%) [11]. However, this conclusion was drawn from a single-rater study, limiting its clinical applicability where multiple raters would evaluate the same patient. In addition, our previous work contradicts this initial report as FMS (CV: 136%) was found to be less repeatable compared to FMD (CV: 25%) [15]. One possible reason for this discrepancy is the heterogeneity in viscoelasticity properties between the upper limb arterial segments used to estimate PWV. Ellins et al., [11] estimated FMS using reactive hyperemia-induced changes in PWV over the brachial to radial arterial segment, which is a muscular arterial segment with thicker tunica media. On the other hand, we measured PWV over a mixed carotid-radial segment where the carotid artery has a higher elastin-to-collagen ratio resulting in a greater distensibility compared to the radial and brachial arteries [15].

From a methodological point of view, it is plausible that regional PWV measurements do not reflect exact responses of arterial stiffness to reactive hyperemia, given the poor agreement between local and regional measures of PWV (carotid PWV vs. carotid-femoral PWV) [16]. Moreover, the measurement of the distance between arterial sites through which PWV travels is the main source of inaccuracy [17–19]. In fact, measurement of travel distances on the surface of the body may not accurately represent the true length and anatomy of the arterial segments, especially in people with obesity, and when arteries become increasingly tortuous with age [20]. To circumvent these issues, mathematical models derived from the Bramwell & Hill equation [21] have been developed to estimate PWV using a single arterial site [22] or local PWV of a single vascular bed [23]. Ultrasound-based methods are increasingly being used to access the local mechanical properties of arterial walls combined with changes in pressure and related volume fluctuations, without the need for a circulatory model [24]. Importantly, local PWV derived from pressure and ultrasound diameter measurements conducted simultaneously with FMD is likely to be more precise in quantifying local artery stiffness responses to reactive hyperemia [23]. This could help clarify the controversial repeatability and relevance of FMS as an alternative method to FMD. Therefore, this study aimed to compare the repeatability of offline ultrasound local estimates of FMS and FMD within (intra-rater) and between different raters (inter-rater).

## Methods

### Participants

Twenty-four apparently healthy and physically active male participants, aged 23–75 years, were recruited for this study. Exclusion criteria included smoking, cardiovascular (e.g., heart failure, coronaropathy), metabolic (e.g., diabetes mellitus), and renal disease. Participants were recruited from the great metropolitan Lisbon area from January to July of 2020. All participants reported to the laboratory in a fasted state (≥ 6h) and refrained from strenuous exercise, vitamin supplements, and foods/drinks containing caffeine, and alcohol ≥ 12h [7]. Inter-day repeatability was assessed on two occasions always at the same time of the day (in the morning) with a minimum of 48h between sessions. Intra-day repeatability was assessed with two measurements performed 20 min apart. All participants gave written informed consent after a detailed explanation of the experimental procedures and aims of the study. All experimental procedures were conducted from January to August of 2022, approved by the ethics committee of Faculdade de Motricidade Humana–Universidade de Lisboa (10/2020), and were aligned with the Declaration of Helsinki for human research.

### Flow-mediated dilation procedures

FMD was assessed in the right brachial artery with an ultrasound equipped with a 7.5-MHz linear array probe incorporating a 5-MHz Doppler transducer (Arietta V60, Hitachi Aloka Medical Ltd, Mitaka-shi, Tokyo, Japan), placed ~4 cm above the antecubital fossa, and held by a mechanical clamp following standard guidelines [7, 25]. Before each measurement, the participants rested in a supine position for 15 min with their right arms extended <80° laterally from the torso and at the heart level, in a quiet climate control room (22-24°C). Reactive hyperemia was induced by rapid cuff deflation following a 5 min forearm occlusion at 250 mmHg. The diameter of the brachial artery was measured with automated edge detection software [7]. Briefly, this validated in-built software of the Arietta V60 ultrasound (eTRACKING, FMD preset, SOP-ARIETTA60-16) allowed for vasomotion tracing during the FMD procedure using calipers placed on near and far walls.

### Offline analysis

This is a secondary analysis of an existing dataset. In a previous publication of this laboratory, we compared the single-rater repeatability of carotid-radial applanation tonometry, FMS and echo-tracking FMD. For the present analysis, only the raw data from the FMD ultrasound scans were used to estimate brachial artery FMS and FMD. Offline analyses were conducted independently by two raters with consecutive inter-day CVs < 5% for brachial diameters and PWV before data collection.

#### Flow-mediated dilation

Using a tailored R script, brachial artery diameter (D_bas_) was trimmed averaged (95%) during the last 60-s of baseline period, and peak diameter (D_peak_) was determined over 10-s time bin averages during the first 3 minutes after cuff-deflation. FMD was calculated as an absolute change ($FMD(mm)={peak\;diameter}_{post―ischemia}-D_{bas}$) and as a relative change ($FMD(\%)=\frac{absolute\;change}{D_{bas}}\times 100\%$) in brachial artery diameter. All image acquisitions and analyses were performed by the same researcher who had more than 100 hours of experience.

#### Flow-mediated slowing

The PWV of the brachial artery was estimated from the raw distensibility and blood pressure signals of the FMD measurement using the manufacturer’s equations (Eq 1 and Eq 2). Systolic and diastolic blood pressures required to estimate beta stiffness and PWV were recorded using beat-by-beat finger plethysmography (Finapres Nova, The Netherlands), and were averaged (95% trimmed mean) over the duration of the FMD measurement. Brachial systolic and diastolic diameters required to estimate PWV were identified offline using a tailored R script. Briefly, this script sorted the rows corresponding to systole and diastole, which were time aligned to the cardiac cycle in the ultrasound FMD raw file, into two separate databases. Then,, PWV was trimmed averaged during the last 60-s of baseline period, whereas min PWV was determined over 10-s time bin averages during the first 3 minutes after cuff-deflation (S1 Fig). FMS was calculated as an absolute-change (${FMS(m.s^{-1})=bPWV}_{post-ischemia}-{bPWV}_{baseline}$) and as an relative change ($FMS(\%)=\frac{absolute\;change}{{PWV}_{baseline}}\times 100\%$) in PWV [11, 26].


$$\beta =\ln\left[\frac{SBP/DBP}{(D_{syst}-D_{diast})/D_{diast}}\right]$$ (Eq 1)

Where, β is beta stiffness; SBP, brachial systolic blood pressure, DBP, diastolic blood pressure; D_syst_, brachial artery diameter during systole; D_diast_, brachial artery diameter during diastole.


$$PWV=\sqrt{\left(\beta \times DBP)/(2\rho \right)}$$ (Eq 2)

Where, PWV is brachial artery pulse wave velocity; β, beta stiffness, DBP, brachial diastolic blood pressure; and ρ, blood density—assumed constant (1050 kg/m^3^).

### Statistics analysis

A power analysis using the R package ICC.Sample.Size [27] with an a priori ICC estimate of 0.80 suggested that 24 participants were required to ensure good in intra and inter-day repeatability of measurements (α = 0.05, 1−β = 0.90, k = 2, null hypothesis = 0.40). This ICC estimate was obtained from the FMD repeatability findings of a multicenter trial that included specialized vascular laboratories, where operators demonstrated CVs < 15% for %FMD [28]. The distributions of PWV and FMS were tested for normality with the Shapiro-Wilk test and plot representation. Repeatability assessment of FMS and FMD analyses within and between raters was conducted using the coefficient of variation (SD/Mean * 100) and two-way absolute agreement mixed models intraclass correlation coefficient (ICC (2,1)) computed with irr package in R [29]. The ICC was interpreted as follows: poor < 0.50, moderate [0.50, 0.74], good [0.75, 0.90], and excellent >0.90 [30] Bland-Altman plots were also used to evaluate the repeatability of FMS and FMD using ggplot 2 package in R [29]. All statistical analyses were conducted using R, version 4.1.0 [31], with a significant level (α) of 0.05.

## Results

The clinical and demographic characteristics of the participants are depicted in Table 1. No significant associations between age and FMS or FMD were observed.

**Table 1:** Characteristics of the participants (n = 24).

Characteristic	
Age (years)	45 (19)
Height (m)	1.7 (0.1)
Weight (kg)	78.5 (9.3)
Body mass index (kg/m^2^)	25.7 (2.4)
Waist circumference (m)	0.91 (0.09)
Fat mass (%)	21.3(6.5)
bSBP (mmHg)	125 (14)
bDBP (mmHg)	76 (11)
HR (b.min^-1^)	60 (8)
% FMD	6.41 (3.41)
% FMS	-7.25 (3.82)
D_bas_ (mm)	4.01 (0.56)
PWV_bas_ (m.s^-1^)	8.58 (0.73)

Data presented as mean (SD). Abbreviations: bSBP, brachial systolic blood pressure; bDBP, brachial diastolic blood pressure; FMD, flow-mediated dilation; D_bas_, resting brachial artery diameter; FMS, flow-mediated slowing; PWV_bas_, brachial artery pulse wave velocity.

### Intra-rater repeatability

Each rater demonstrated excellent intra-day repeatability for %FMS and %FMD measurements (ICCs > 0.95 and CVs < 15%). Intra-rater repeatability was not different between FMS or FMD measurements as perceived by the overlap of 95%CIs of the ICCs (Table 2).

**Table 2:** Intra-rater repeatability statistics for intra-day and inter-day analyses.

	CV		ICC (95%CI)			Bias (95% LOA)		SD	
	Rater 1	Rater 2	Rater 1	Rater 2		Rater 1	Rater 2	Rater 1	Rater 2
*Intra day*									
FMD (%)	14	12	0.97 (0.92 to 0.99)	0.97 (0.86 to 0.99)		0.43 (-1.91 to 2.77)	0.69 (-1.04 to 2.42)	1.19	0.70
FMD (mm)	16	14	0.86 (0.65 to 0.98)	0.94 (0.76 to 0.98)		0.04 (-0.12 to 0.19)	0.04 (-0.06 to 0.13)	0.08	0.05
FMS (%)	12	15	0.95 (0.89 to 0.98)	0.93 (0.84 to 0.97)		0.16 (-3.16 to 3.48)	0.62 (-3.00 to 4.22)	1.69	1.83
FMS (m.s^-1^)	13	17	0.95 (0.88 to 0.98)	0.90 (0.77 to 0.96)		0.05 (-0.30 to 0.36)	0.05 (-0.40 to 0.51)	0.17	0.23
D_bas_ (mm)	2	2	0.99 (0.97 to 0.99)	0.98 (0.96 to 0.99)		0.02 (-0.23 to 0.27)	0.03 (-0.28 to 0.33)	0.13	0.15
D_peak_ (mm)	3	2	0.98 (0.96 to 0.99)	0.98 (0.95 to 0.99)		0.05 (-0.23 to 0.34)	0.06 (-0.26 to 0.38)	0.14	0.16
PWV_bas_ (m.s^-1^)	3	3	0.90 (0.72 to 0.96)	0.92 (0.68 to 0.97)		-0.24 (-1.13 to 0.66)	-0.27 (-0.98 to 0.44)	0.46	0.36
PWV_min_ (m.s^-1^)	3	3	0.90 (0.74 to 0.96)	0.92 (0.74 to 0.97)		- 0.20 (-1.01 to 0.61)	-0.21 (-0.88 to 0.46)	0.42	0.34
*Inter-day*									
FMD (%)	14	18	0.90 (0.78 to 0.96)		0.85 (0.64 to 0.94)	0.27 (-3.82 to 4.36)	0.60 (-4.37 to 5.58)	2.09	2.54
FMD (mm)	15	14	0.84 (0.63 to 0.93)		0.83 (0.60 to 0.92)	0.01 (0.17 to 0.19)	0.02 (0.18 to 0.21)	0.09	0.10
FMS (%)	21	23	0.76 (0.44 to 0.91)		0.70 (0.34 to 0.87)	-1.03 (-6.60 to 4.52)	-0.49 (-5.52 to 5.63)	2.83	3.12
FMS (m.s^-1^)	24	25	0.70 (0.32 to 0.87)		0.68 (0.28 to 0.84)	-0.10 (-0.67 to 0.47)	0.05 (-0.40 to 0.51)	0.29	0.23
D_bas_ (mm)	3	3	0.94 (0.83 to 0.98)		0.94 (0.81 to 0.98)	-0.13 (-0.59 to 0.32)	-0.14 (-0.61 to 0.33)	0.23	0.24
D_peak_ (mm)	4	3	0.94 (0.82 to 0.99)		0.94 (0.83 to 0.98)	-0.12 (-0.60 to 0.35)	-0.12 (-0.61 to 0.37)	0.23	0.23
PWV_bas_ (m.s^-1^)	4	4	0.78 (0.50 to 0.91)		0.74 (0.40 to 0.89)	0.08 (-1.23 to 1.41)	0.02 (-1.38 to 1.42)	0.67	0.72
PWV_min_ (m.s^-1^)	4	4	0.85 (0.66 to 0.94)		0.83 (0.61 to 0.92)	-0.02 (-1.05 to 1.02)	-0.03 (-1.12 to 1.05)	0.53	0.55

Abbreviations: FMD: flow-mediated dilation; D_bas_, brachial artery baseline diameter; D_peak_, brachial artery peak diameter; FMS: flow-mediated slowing; PWV_min_, minimum value of brachial artery pulse wave velocity; PWV_bas_, baseline brachial artery pulse wave velocity.

**Fig 1:**
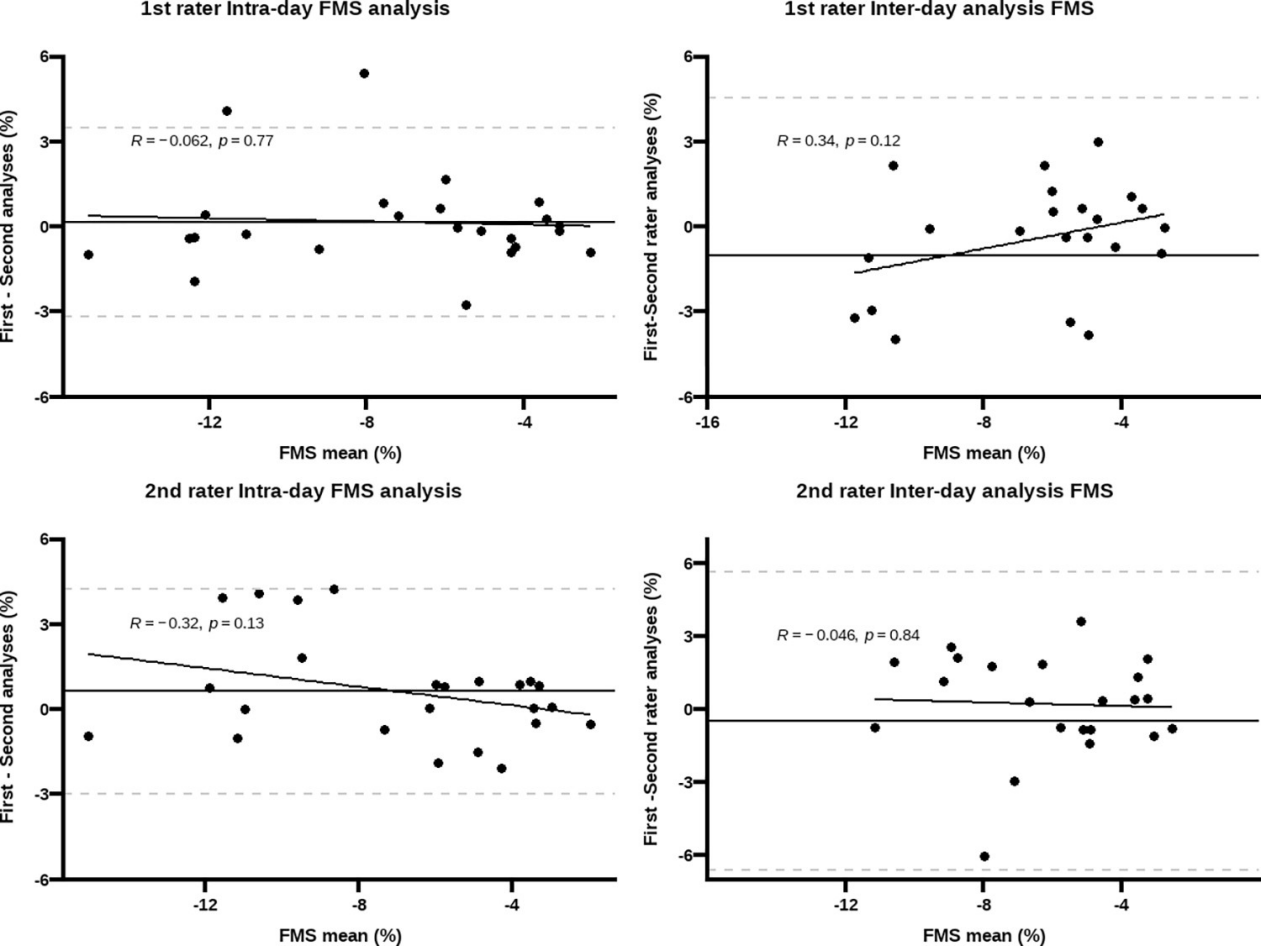
Bland Altman plots for intra-rater agreement between repeated measurements of flow-mediated slowing (FMS). Y-axis represents the difference between the first and second analysis; the dashed grey lines correspond to the 95%LOA and the black line represents the bias. No proportional bias was observed given the non-significant associations between bias and magnitude of differences *(p* >0.05).

**Fig 2:**
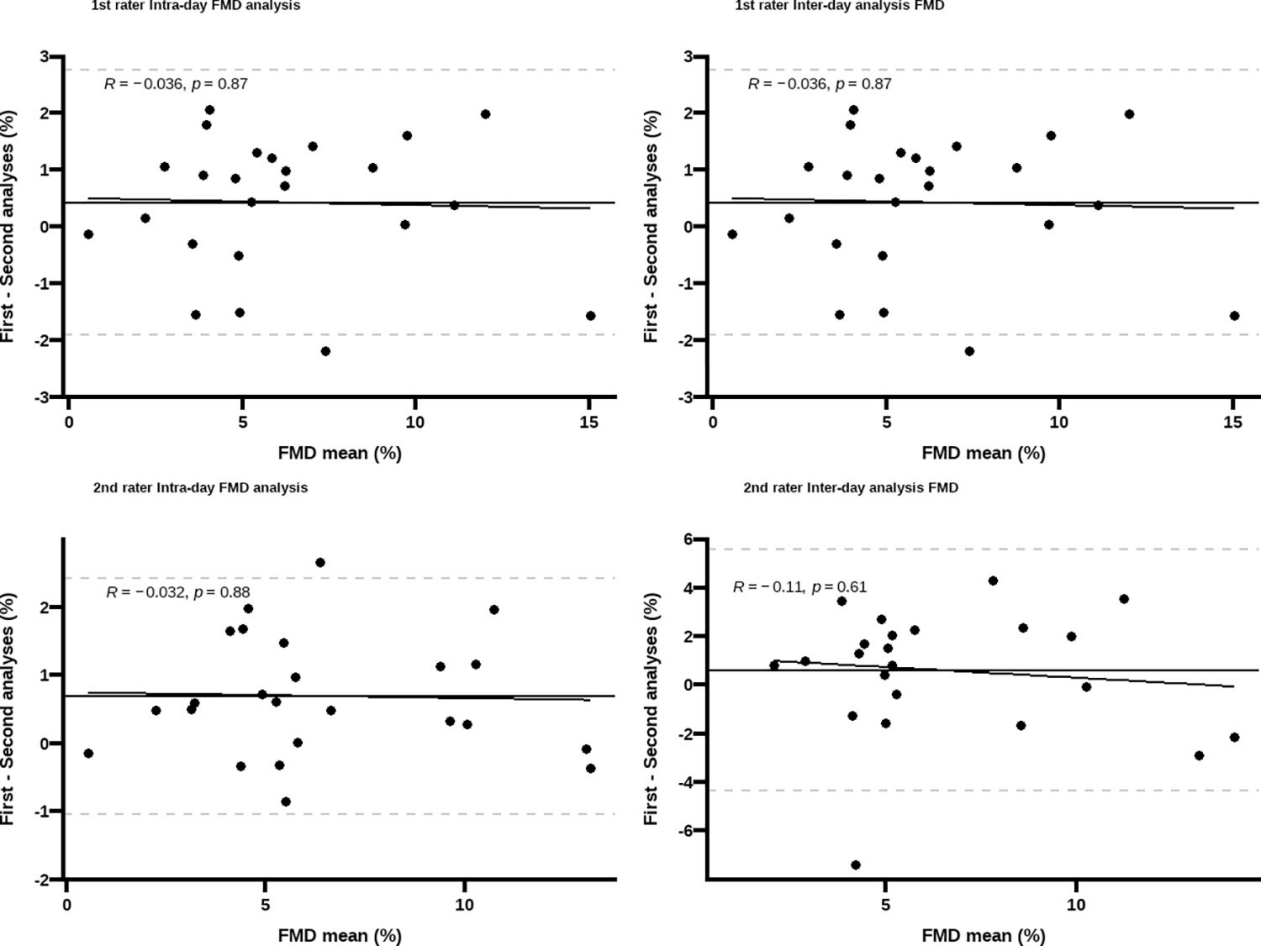
Bland Altman plots flow-mediated dilation (FMD) intra-rater agreement. Y-axis represents the difference between the first and second analysis; the dashed grey lines correspond to the 95%LOA and the black line represents the bias. No proportional bias was observed given the non-significant associations between bias and magnitude of differences (*p* >0.05).

All parameters used to estimate FMS and FMD exhibited excellent repeatability among raters (CVs < 5%). Bland Altman plots did not show evidence of proportional bias and only repeated measurements from two participants in FMS, and one participant in FMD, fell outside the 95% LOA (Figs 1 and 2).

Inter-day repeatability was not different between raters in repeated measurements of FMD or FMS, but the latter showed poorer repeatability (FMS: CV ~ 22%, ICC ~ 0.74 vs. FMD: CV ~ 16%, ICC ~ 0.88). Similarly, in intra-day repeatability, all parameters used to estimate FMS and FMD showed excellent repeatability (Table 3). The Bland Altman plots did not show evidence of proportional bias and only repeated measurements from two participants in FMS and one in FMD, fell outside the 95% LOA (Figs 1 and 2).

**Table 3:** Inter-rater repeatability statistics for intra-day (1st and 2nd) and inter-day analyses.

	CV	ICC (95%CI)	Bias (95% LOA)	SD
*Intra day*				
FMD (%)				
1^st^	9	0.97 (0.94 to 0.99)	-0.35 (-2.51 to 1.80)	1.10
2^nd^	12	0.95 (0.90 to 0.98)	-0.09 (-2.92 to 2.73)	1.44
FMD (mm)				
1^st^	10	0.94 (0.86 to 0.97)	-0.01 (-0.12 to 0.11)	0.06
2^nd^	12	0.92 (0.82 to 0.97)	-0.01 (-0.13 to 01.2)	0.06
FMS (%)				
1^st^	15	0.91 (0.78 to 0.96)	-0.63 (-4.73 to 3.49)	2.10
2^nd^	15	0.92 (0.82 to 0.97)	-0.16 (-4.37 to 4.05)	2.15
FMS (m.s^-1^)				
1^st^	17	0.86 (0.68 to 0.94)	-0.05 (-0.55 to 0.45)	0.25
2^nd^	18	0.92 (0.83 to 0.97)	-0.03 (-0.43 to 0.37)	0.21
D_bas_ (mm)				
1^st^	1	0.99 (0.98 to 0.99)	0.01 (-0.13 to 0.14)	0.07
2^nd^	1	0.99 (0.97 to 0.99)	0.01 (-0.25 to 0.27)	0.13
D_peak_ (mm)				
1^st^	1	0.99 (0.98 to 0.99)	-0.01 (-0.17 to 0.16)	0.08
2^nd^	2	0.99 (0.97 to 0.99)	0.01 (-0.32 to 0.32)	0.16
PWV_bas_ (m.s^-1^)				
1^st^	1	0.98 (0.96 to 0.99)	0.07 (-0.34 to 0.49)	0.21
2^nd^	1	0.98 (0.96 to 0.98)	0.04 (-0.34 to 0.43)	0.20
PWV_min_ (m.s^-1^)				
1^st^	1	0.97 (0.92 to 0.99)	0.02 (-0.52 to 0.57)	0.28
2^nd^	1	0.98 (0.95 to 0.99)	0.01 (-0.36 to 0.38)	0.19
*Inter-day*				
FMD (%)	7	0.99 (0.98 to 0.99)	-0.02 (-1.26 to 1.22)	0.63
FMD (mm)	7	0.98 (0.97 to 0.99)	-0.01 (-0.06 to 0.06)	0.03
FMS (%)	11	0.90 (0.76 to 0.96)	-0.08 (-3.35 to 3.18)	1.66
FMS (m.s^-1^)	11	0.85 (0.65 to 0.93)	0.10 (-0.60 to 0.80)	0.36
D_bas_ (mm)	1	0.99 (0.98 to 0.99)	-0.01 (-0.12 to 0.11)	0.06
D_peak_ (mm)	1	0.99 (0.98 to 0.99)	-0.01 (-0.15 to 0.13)	0.07
PWV_bas_ (m.s^-1^)	1	0.99 (0.98 to 0.99)	0.01 (-0.13 to 0.16)	0.07
PWV_min_ (m.s^-1^)	1	0.98 (0.97 to 0.99)	0.01 (-0.30 to 0.30)	0.15

Abbreviations: 1^st^: first analysis; 2^nd^: second analysis; FMD, flow-mediated dilation; D_bas_, brachial artery baseline diameter; D_peak_, brachial artery peak diameter; FMS, flow-mediated slowing; PWV_min_, minimum value of pulse wave velocity; PWV_bas_, pulse wave velocity baseline

### Inter-rater repeatability

Intra-and inter-day analyses of repeated measurements of %FMS and %FMD, as well as calculation parameters (e.g., D_bas_, PWV_bas_), showed excellent inter-rater repeatability with ICCs > 0.90 and CVs < 15% (Table 3). The inter-rater repeatability for %FMD (ICC:0.99, 95% CI: 0.98 to 0.99) was better compared to %FMS (ICC: 0.85, 95% CI: 0.65 to 0.93) for inter-day analyses.

**Fig 3:**
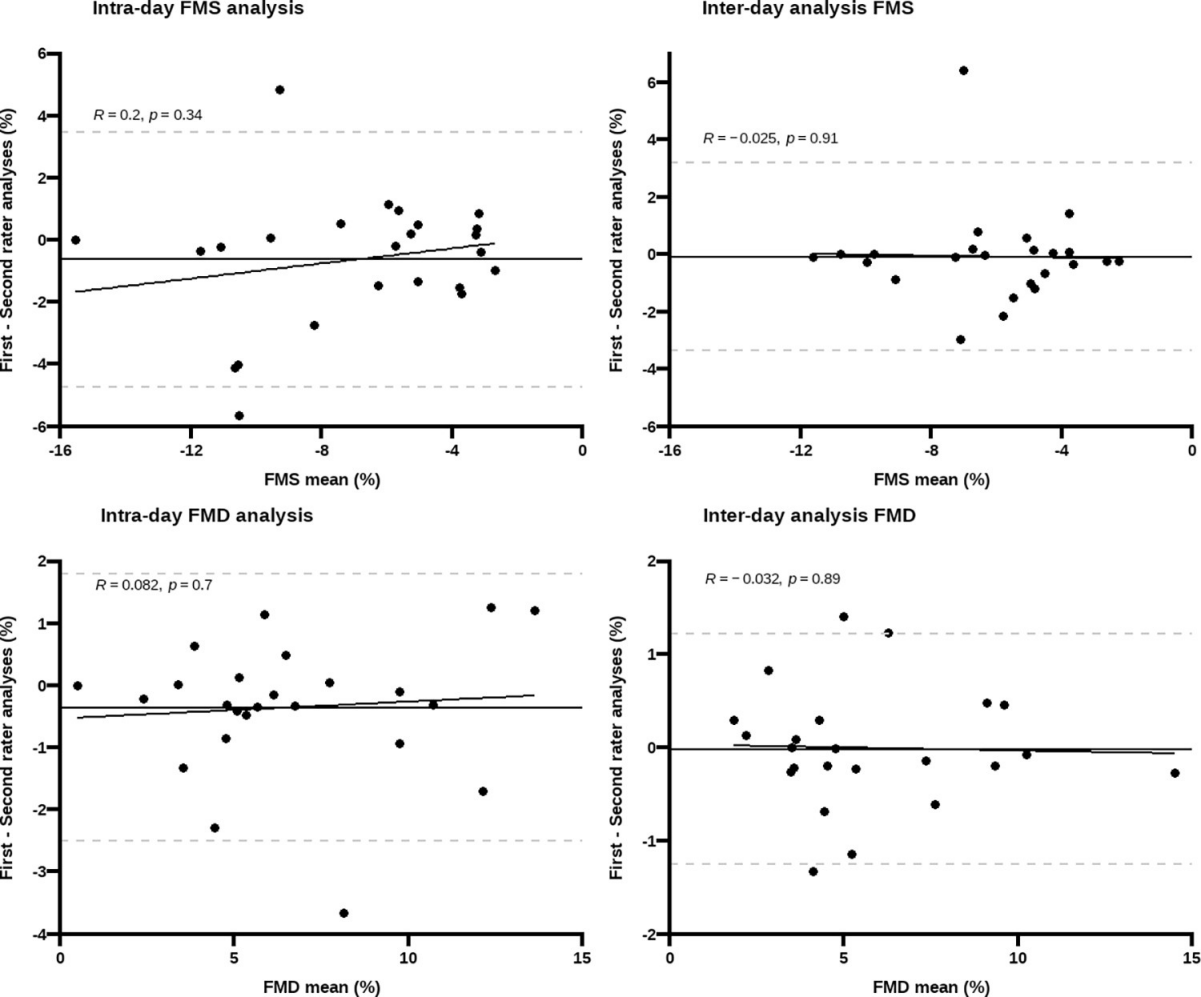
Bland Altman plots for inter-rater agreement between repeated measurements of flow-mediated slowing (FMS) and flow-mediated dilation (FMD). Y-axis represents the difference between the first and second analysis; the dashed grey lines correspond to the 95%LOA and the black line represents the bias. No proportional bias was observed given the non-significant associations between bias and magnitude of differences *(p* >0.05).

Bland-Altman plots for repeated measurements of %FMS and %FMD displayed excellent inter-rater repeatability in intra and inter-day analyses, with no evidence of bias (close to zero) or proportionality in bias (Fig 3, Table 3). From visual plot inspection, a maximum of two participants fell outside the 95% LOA for both %FMD and %FMS. Still, inter-rater analyses of FMS showed wider 95% LOA and SD of bias compared to FMD.

## Discussion

This study aimed to examine the repeatability of FMS using ultrasound local estimates of PWV and FMD of the brachial artery, within (intra-rater) and between different raters (inter-rater). To our knowledge, this study was the first to show that intra- and inter-rater offline analyses of local FMS and FMD measurements show excellent repeatability.

### Intra-rater repeatability

Both raters demonstrated good to excellent intra- and inter-day repeatability in the offline analyses of local estimates of FMS and FMD [7, 32]. These findings are contradictory to those from regional FMS (CV > 100%) published earlier by this research group [15], but aligned with the FMS repeatability reported by Ellins et al. [11]. Together, this suggests that the heterogeneity in viscous-elastic properties of the arteries is likely the main confounder in regional FMS including both elastic and muscular arteries. Thus, using local estimates of PWV to calculate FMS is advantageous as it reflects exact responses of brachial artery stiffness to reactive hyperemia and reduces both methodological (e.g., distance measurement) and physiological (e.g., vascular viscous-elastic heterogeneity) confounders inherent in regional PWV measurements. In this study, consistent decelerations to reactive hyperemia were observed in local PWV that we did not observe in regional PWV [15]. Hypothetically, this supports the idea that increases in NO-mediated vasodilation mediated by shear stress underpin local but not regional carotid radial PWV reductions to reactive hyperemia. Therefore, these confounding factors can explain the equivocal repeatability of FMS measurements between studies [11, 15] and are likely to extend to other devices used in FMS repeatability studies.

### Inter-rater repeatability

An often-overlooked consideration dictating the use of a method in clinical settings is the agreement between raters. In this study, the inter-rater repeatability of FMS and FMD was excellent, which is in line with previous work that evaluated the repeatability of offline analysis of FMD [33]. To our knowledge, this is the first study to show that offline analyses of local estimates of FMS and FMD are highly repeatable using a tailored built R-script based on FMD guidelines and the Bramwell & Hill derived equations to estimate brachial PWV deceleration to reactive hyperemia [7, 21, 34, 35]. Thus, this might represent an inexpensive alternative since R is open-source software to conduct FMD and FMS offline analyzes. However, these results also suggest that the main source of measurement error does not come from the offline analysis of FMD and FMS, but rather from technical challenges during the measurement. This reinforces the need for extensive operator training and standardization of FMD or FMS procedures [36].

### Is flow-mediated slowing relevant?

FMS derived from regional PWV has been suggested to be useful as a peripheral marker of endothelial function suitable for the evaluation of large-scale populations and early-stage disease cohorts due to its simplicity and apparent superior repeatability compared to FMD [11]. However, estimating FMS based on reactive hyperemia-induced changes on local brachial artery PWV disputes this rationale. In fact, this methodological approach uses similar methodology and technology as FMD. Based on present findings, FMS should be considered as a complement [11], rather than an alternative [26], to the FMD procedure until future research clarifies: 1) the repeatability of FMS estimated using regional PWV; 2) the underlying mechanisms of the reactive hyperemia induced changes in regional PWV; 3) and the prognostic value of FMS for cardiovascular disease and events.

### Limitations

This study is not without limitations. The FMS was estimated based on the local brachial PWV derived from the raw signals of the FMD measurement. Thus, the known limitations of the FMD procedure also apply to those of FMS. In fact, the intra-rater repeatability over measurements performed in different days for local FMS was poorer than that for FMD, which is probably the result of multistep error propagation, first from local estimations of PWV from distensibility signals, and secondly from the FMS ratio calculation [37]. Second, the endothelium-independent vasodilation in response to sublingual glyceryl trinitrate was not evaluated. Thus, we cannot ascertain the contribution of smooth muscle cells to PWV response to reactive hyperemia. Furthermore, this study design precludes any explanation of the effects of differential mechanisms on the equivocal repeatability of the FMS estimated using regional PWV. Third, only healthy male individuals were sampled. The repeatability of measurements of FMD and FMS may be lower in participants with cardiovascular risk factors and chronic diseases [7]. Moreover, we cannot rule out the possibility of sex differences concerning the repeatability of FMD and FMS and different FMS response patterns.

### Conclusions

Our study demonstrated that offline analyses of local estimates of FMS and FMD were repeatable within and between different raters. Consistent local PWV decelerations, estimated through ultrasound distensibility signals, suggest that FMS is dependent on the vascular beds evaluated, but dispute the ease of use and the usefulness of FMS.

## Supporting information

S1 Fig Examples of ultrasound signals smoothing conducted offline in R for flow-mediated slowing (FMD) and flow-mediated dilation (FMD). Panel A depicts an example of brachial pulse wave velocity (PWV) deceleration to reactive hyperemia–FMS—following forearm occlusion. PWV was estimated using manufacturer’s formulas. Panel B depicts an example of brachial artery dilation to reactive hyperemia–FMD—following supra systolic forearm occlusion.
(DOCX)

S1 Dataset (XLSX)

## List of abbreviations

FMS: Flow-mediated slowing
FMD: Flow-mediated dilation
PWV: Pulse wave velocity
NO: nitric oxide
CV: coefficient of variation
ICC: intraclass correlation coefficient
D_bas_: brachial artery baseline diameter
D_peak_: brachial artery peak diameter
SBP: systolic blood pressure
DBP: diastolic blood pressure
β: beta stiffness
