# Supplementary figures and images for: Intra and inter-rater repeatability of brachial artery ultrasound estimates of flow-mediated slowing and flow-mediated dilation
Source: PLoS One. 2023 Jun 28;18(6):e0287759. doi: 10.1371/journal.pone.0287759 (PMC10306196; doi:10.1371/journal.pone.0287759)

Supplement


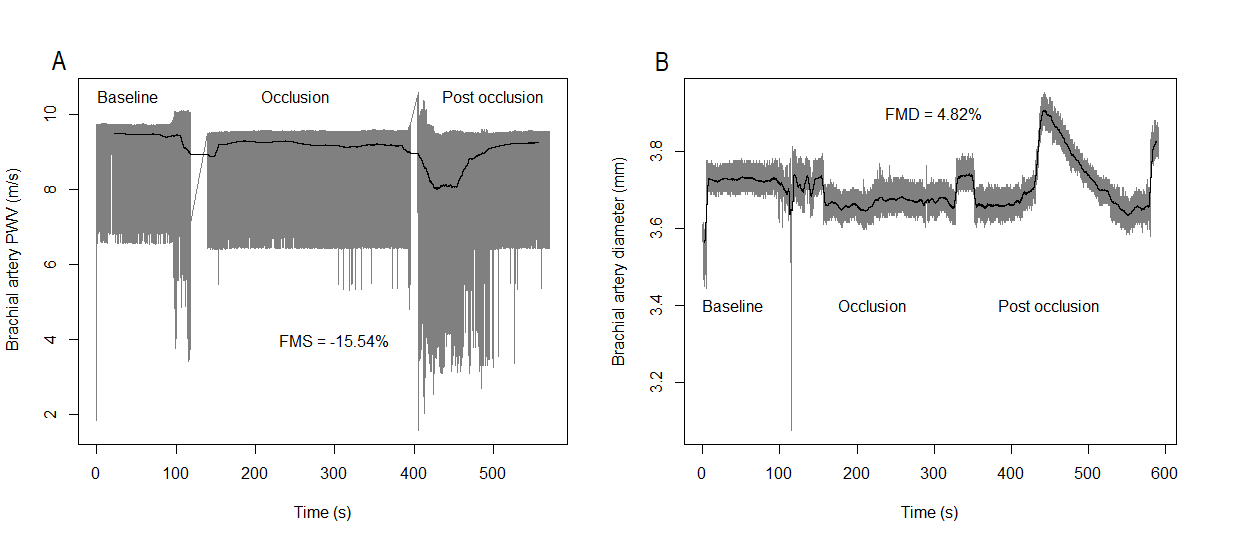

Supplement: S1 Fig — Panel A depicts an example of brachial pulse wave velocity (PWV) deceleration to reactive hyperemia–FMS—following forearm occlusion. PWV was estimated using manufacturer’s formulas. Panel B depicts an example of brachial artery dilation to reactive hyperemia–FMD—following supra systolic forearm occlusion. (DOCX) [file pone.0287759.s001.docx]
